# Efficient Intravenous Tumor Targeting Using the αvβ6 Integrin-Selective Precision Virotherapy Ad5_NULL_-A20

**DOI:** 10.3390/v13050864

**Published:** 2021-05-08

**Authors:** James A. Davies, Gareth Marlow, Hanni K. Uusi-Kerttula, Gillian Seaton, Luke Piggott, Luned M. Badder, Richard W. E. Clarkson, John D. Chester, Alan L. Parker

**Affiliations:** 1 Division of Cancer and Genetics, Cardiff University School of Medicine, Cardiff CF14 4XN, UK; DaviesJA9@Cardiff.ac.uk (J.A.D.); gmarlow@hotmail.com (G.M.); hanni.kerttunen@gmail.com (H.K.U.-K.); badderlm@cardiff.ac.uk (L.M.B.); ChesterJD@Cardiff.ac.uk (J.D.C.); 2 School of Biosciences, Cardiff University, Cardiff CF24 4HQ, UK; SeatonG1@Cardiff.ac.uk (G.S.); Luke.piggott@debiopharm.com (L.P.); ClarksonR@Cardiff.ac.uk (R.W.E.C.); 3 Velindre Cancer Centre, Cardiff CF14 2TL, UK

**Keywords:** adenovirus, oncolytic, virotherapy, targeting, αvβ6 integrin, systemic delivery

## Abstract

We previously developed a refined, tumor-selective adenovirus, Ad5_NULL_-A20, harboring tropism ablating mutations in each major capsid protein, to ablate all native means of infection. We incorporated a 20-mer peptide (A20) in the fiber knob for selective infection via αvβ6 integrin, a marker of aggressive epithelial cancers. **Methods:** To ascertain the selectivity of Ad5_NULL_-A20 for αvβ6-positive tumor cell lines of pancreatic and breast cancer origin, we performed reporter gene and cell viability assays. Biodistribution of viral vectors in mice harboring xenografts with low, medium, and high αvβ6 levels was quantified by qPCR for viral genomes 48 h post intravenous administration. **Results:** Ad5_NULL_-A20 vector transduced cells in an αvβ6-selective manner, whilst cell killing mediated by oncolytic Ad5_NULL_-A20 was αvβ6-selective. Biodistribution analysis following intravenous administration into mice bearing breast cancer xenografts demonstrated that Ad5_NULL_-A20 resulted in significantly reduced liver accumulation coupled with increased tumor accumulation compared to Ad5 in all three models, with tumor-to-liver ratios improved as a function of αvβ6 expression. **Conclusions:** Ad5_NULL_-A20-based virotherapies efficiently target αvβ6-integrin-positive tumors following intravenous administration, validating the potential of Ad5_NULL_-A20 for systemic applications, enabling tumor-selective overexpression of virally encoded therapeutic transgenes.

## 1. Introduction

Cancer virotherapies are emerging in the clinical setting, with oncolytic viral therapy being an approved form of immunotherapy since talimogene laherparepvec (T-VEC, Imlygic^®^) was approved by the Food and Drug Administration (FDA) and European Medicines Agency (EMA) for melanoma treatment in 2015 [1]. Moreover, an accumulating body of evidence indicates that the immunogenic nature of oncolytic-virus-induced cell death provides a suitable environment to sensitize resistant tumors to immunotherapies [2]. Various oncolytic viruses (OVs), such as HF10 (Canerpaturev—C-REV) and CVA21 (CAVATAK), are now actively being developed in phase II as monotherapies, or in combination with immune checkpoint inhibitors against melanoma [3].

OVs have shown efficacy when delivered by direct intratumoral injection. However, systemic delivery of OV has proven more challenging. For OVs to treat a range of primary and metastatic tumors, they will need to be efficiently delivered via intravenous administration [4]. This will require the development of new OVs designed to overcome the problems facing systemic administration.

Adenoviruses (Ad) have proven popular choices for oncolytic applications. Their double-stranded DNA genome is ideally suited to genetic manipulation, and they can be grown to high, clinically useful titers. Adenovirus 5 (Ad5) is the most commonly used platform for cancer and other gene therapy applications [5]; however, this serotype has several features that may hamper its use clinically as an oncolytic agent.

There have been a large number of clinical trials involving Ad5-based oncolytic vectors [6]. Onyx-15 is the most widely studied and has been the subject of 18 phase I and II clinical trials with published results [7]. Initial results from intertumoral injections of solid tumors were promising [8,9]; however, definite antitumor efficacy could not be demonstrated. Subsequent trials of systemic injection also demonstrated poor efficacy [10,11], and low levels of the virus were detected in the tumor compared to the liver and spleen [11]. Clearly, improvements must be made to allow Ad5-based oncolytics to efficiently target tumors.

In vitro Ad5 enters host cells via an initial interaction between viral fiber protein and its primary receptor, coxsackie and adenovirus receptor (CAR) [12]. Subsequently, Ad5 internalizes via αvβ3/5 integrins mediated by the viral penton base protein [13]. CAR is ubiquitously expressed within tight junctions on polarized epithelial cells but is often downregulated in cancers [14], making it a poor target for cancer therapies. CAR is present on the surface of human erythrocytes, providing an Ad5 sequestration mechanism that protects against systemic infection [15,16]. Complement receptor (CR1), which is also expressed by human erythrocytes, binds Ad5 in the presence of antibodies and complements and is able to inhibit systemic infection [15]. These receptors are not found on murine erythrocytes, and this may explain some of the inconsistencies found between human and animal models. Ad5 is a common respiratory virus, with seroprevalence rates close to 100% in certain populations [17]. Neutralizing antibodies (nAbs) can rapidly inactivate therapeutic vectors. In addition, there is extensive off-target sequestration to the liver upon systemic administration via bridging of the viral hexon protein to heparan sulphate proteoglycans (HSPGs) [18] via human coagulation factor 10 (FX) [19].

We previously ablated all the native tropisms of Ad5 by introducing a panel of point mutations in the main capsid components [20]. This generated a triple detargeted Ad5-based vector containing a combination of tropism modifications in hexon hypervariable region 7 (HVR7 mutation) [21], fiber knob AB loop (KO1 mutation) [22], and penton integrin-binding motif Arg-Gly-Asp (RGD -> RGE mutation) [13]. The vector was made cancer-selective by genetic incorporation of an αvβ6 integrin-binding peptide (A20, NAVPNLRGDLQVLAQKVART) within the viral fiber knob HI loop [23]. A20 peptide was originally derived from foot-and-mouth disease virus (FMDV) capsid protein VP1 and has a very high affinity to its native receptor, αvβ6 integrin [24]. αvβ6 integrin represents an exciting candidate for targeted delivery of cancer therapeutics, since it is absent in the normal epithelia but plays a key role in TGF-β-mediated epithelial to mesenchymal transformation (EMT) and metastasis [25,26]. Expression of αvβ6 integrin has been shown to correlate with poor clinical prognosis [27,28], and several advanced therapies are under development that are targeted to this tumor-associated antigen [29,30,31]. This new vector, Ad5_NULL_-A20, was able to selectively and efficiently infect αvβ6-integrin-positive cell lines and primary ovarian tumor cells. Furthermore, our in vivo biodistribution analysis demonstrated significantly reduced sequestration in “off-target” organs compared to the unmodified parental vector, Ad5. In our in vivo efficacy studies, intraperitoneal administration of oncolytic Ad5_NULL_-A20 resulted in selective and efficient infection of peritoneal tumor metastases in a mouse model of advanced ovarian cancer, resulting in dramatically improved survival rates compared to mice treated with vector controls or with untreated mice [20].

In this study, we have evaluated the potential for Ad5_NULL_-A20 to transduce and kill αvβ6-integrin-positive cancer cells of pancreatic and breast origin. We also sought to establish whether the Ad5_NULL_-A20 platform could selectively infect αvβ6-integrin-positive tumors following intravascular delivery in an in vivo mouse model of breast cancer.

## 2. Materials and Methods

### 2.1. Viruses

All replication-deficient and oncolytic vectors are based on a wild type Ad5 genome captured in a bacterial artificial chromosome (BAC). Subsequent modifications were introduced into the BACs by homologous recombineering [32]. Replication-deficient vectors carry a complete *E1/E3* gene deletion. Oncolytic vectors have a 24-base pair deletion *dl922*‒*947* (Δ24) [33] in the *E1A* gene to restrict viral replication to pRB-defective cells and a T1 mutation [34] in the *E3/19K* gene to enhance oncolytic efficacy. The A20 peptide sequence (NAVPNLRGDLQVLAQKVART) from FMDV was inserted into the fiber knob HI loop. Control virus was produced in HEK293 cells, the Ad5_NULL_-A20 was produced in HEK293-β6 cells. Virus was purified and characterized according to standard protocols [23].

### 2.2. Cell Lines

Pancreatic cell lines used in this study, as well as the triple negative breast cancer cell line BT-20, were kindly gifted by collaborators. BT-474, MDA-MB-231 and MDA-MB-231 were purchased from American Type Culture Collection (ATCC). All cell lines were cultured to ATCC guidelines.

### 2.3. In Vitro Assays

To quantify cell surface receptors, cells were detached with trypsin/EDTA resuspended and incubated on ice for 1 h with the respective primary mouse mAb; anti-CAR (RmcB, Millipore, Watford, UK) and anti-αvβ6 (10D5, Millipore). Bound antibodies were detected with secondary goat antimouse IgG conjugated to Alexa 647 (A21237, LifeTechnologies, Warrington, UK) for 1 h. Cells were analyzed on a BD Accuri C6 (BD Biosciences, Wokingham, UK) flow cytometer. The results were analyzed using the BD Accuri software.

Cell transduction efficiency was assessed in luciferase reporter assays, 20,000 cells were seeded into each well of a 96 well plate and incubated overnight at 37 °C. Cells were infected with 5000 viral particles per cell (vp/cell) in triplicate for 3 h in serum-free media. 48 h post infection, the cells were lysed and analyzed using the Luciferase Assay system (Promega, Southampton, UK) following the manufacturer’s protocol to determine relative light units (RLU). Protein concentration for each well was determined using the BCA assay (Thermo Fisher Scientific, Newport, UK) following the manufacturers protocol. RLU values for each sample were normalized against total protein for each sample (RLU/mg).

Cytotoxicity of oncolytic virus was determined using the CellTiter 96 AQueous One Solution Cell Proliferation assay (Promega) according to the manufacturer’s recommended protocol. 20,000 cells were seeded into each well of a 96 well plate and incubated overnight. Cells were infected with 5000 vp/cell for 3 h in serum-free media. Viable cells were determined at 24, 48, 72, 96, and 144 h after infection, by adding 20 µL CellTiter 96 AQueous One Solution reagent per well. Absorbance was measured at 490 nm after a 2 h incubation in a humidified 5% CO_2_ atmosphere. The percentage of viable cells was calculated relative to untreated cells. Results are mean, *n* = 3, error bars represent standard deviation.

### 2.4. In Vivo Studies

Two patient-derived xenograft (PDX) models [35] of breast cancer, available in-house, which had low (PDX2665) or med/high (PDX3204) levels of αvβ6 integrin expression, as well as a BT-20 (αvβ6^HIGH^) xenograft model (implanted with Matrigel to support growth), were implanted subcutaneously in NSG mice. To determine αvβ6 levels in the PDXs, RNA was extracted using RNAeasy kit, and TaqMan gene expression assay was performed. When tumors reached a palpable size, mice were injected intravenously with 1 × 10^11^ vp of replication deficient Ad5 or Ad5_NULL_-A20. Forty-eight hours postinjection, organs were harvested, and qPCR for viral genomes was performed on DNA isolated from the liver and tumors. Viral and total genomic DNA was obtained using DNeasy Blood & Tissue DNA extraction kit. DNA was subjected to fluorogenic quantitative PCR using Fast SYBR Green Master Mix system in triplicate, using primers for the hexon: Forward: 5′-CGCGGTGCGGCTGGTG-3′ and Reverse: 5′-TGGCGCATCCCATTCTCC-3′. Total adenoviral genomes were calculated using a standard curve of 10^1^–10^7^ viral genomes.

### 2.5. Statistical Analyses

All figures and statistical analyses were done in GraphPad Prism 6.03. Vector transduction efficiency and in vivo biodistribution were analyzed by two-tailed unpaired *t*-tests. * *p* < 0.05; ** *p* < 0.01; *** *p* < 0.001; **** *p* < 0.0001.

## 3. Results

To assess the potential of Ad5_NULL_-A20 as an agent to treat pancreatic and breast cancer, we determined the expression levels of αvβ6 integrin and CAR on a panel of cell lines.

**Figure 1:**
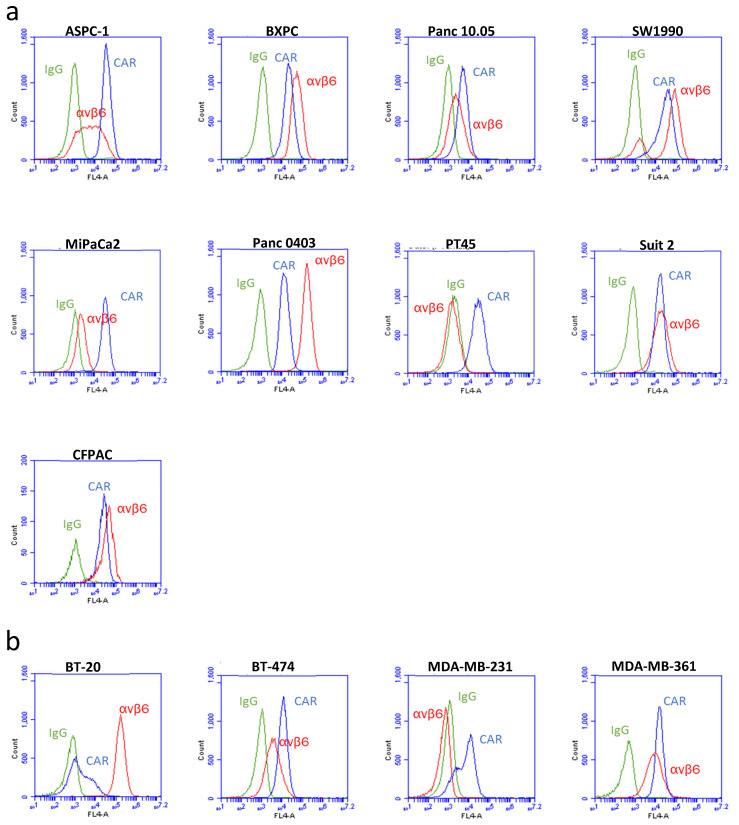
**Expression of αvβ6 integrin and CAR were measured by flow cytometry.** Panels of pancreatic cell lines (**a**) and breast cancer cell lines (**b**) were stained for surface expression of αvβ6 integrin (red), CAR (blue) and IgG control (green). Cells were gated to exclude dead cells, and a minimum of 10,000 events were recorded.

In the nine pancreatic cancer cell lines tested, seven were positive for αvβ6 integrin expression, including BxPc, PANC0403, Suit2, CFPAC, SW1990, PANC10.05, and ASPC-1. Two lines, MiPaCa2 and PT-45 (Figure 1a), were extremely low or negative for αvβ6 expression, respectively. The highest expression levels were seen in PANC0403. CAR was detected in all nine cell lines. We screened four breast cancer cell lines (Figure 1b), and identified 3 cell lines (BT-20, MDA-MB-361, BT474) that expressed αvβ6 integrin and one (MDA-MB-231) that did not express αvβ6. Notably, the cell line BT20 was αvβ6^high^/CAR^null,^ whilst MDA-MB-231 cells were of the opposite phenotype, αvβ6^null^/CAR^high^ (Figure 1b).

**Figure 2:**
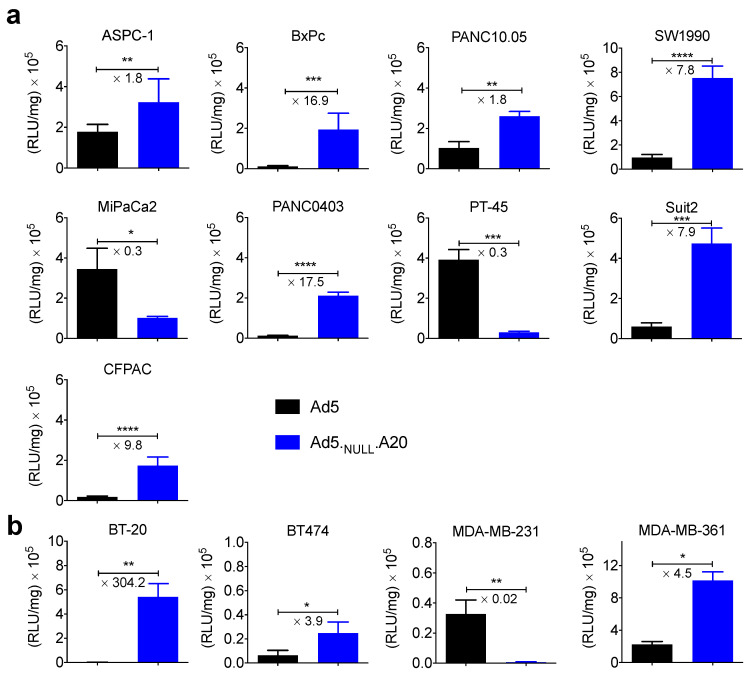
**Transduction of pancreatic and breast cancer cell lines with Ad5 and Ad5_NULL_-A20.** Nine pancreatic cell lines (**a**) and four breast cancer cell lines (**b**) were transduced with 5000 vp/cell of either Ad5 (black bars) or Ad5_NULL_-A20 (blue bars) vectors expressing luciferase. Luciferase expression was quantified 48 h postinfection and normalized to total cellular protein. Error bars represent standard deviation of *n* = 4. (* *p* < 0.05; ** *p* < 0.01; *** *p* < 0.001; **** *p* < 0.0001).

The transduction efficiency of replication-deficient Ad5 and Ad5_NULL_-A20 vectors expressing a luciferase transgene was then assessed in these cell lines (Figure 2).

In all cases, transduction correlated well with the expression levels of αvβ6 integrin/CAR. Cell lines expressing αvβ6 integrin were efficiently and selectively transduced using the Ad5_NULL_-A20 vector. Conversely, cells negative for αvβ6 were poorly transduced by the Ad5_NULL_-A20 vector. The majority of cell lines tested expressed CAR, and these could be transduced by Ad5, where cells expressed αvβ6 integrin and CAR transduction was greater for Ad5_NULL_-A20. Suit2 and MDA-MB-361 cell lines expressed high levels of both αvβ6 and CAR, but transduction of Ad5_NULL_-A20 was 7.9 and 4.6 times greater, respectively. The greatest difference in transduction between Ad5 and Ad5_NULL_-A20 was seen in αvβ6^high^/CAR^null^ BT-20 cells, which showed an increase in transduction of over 300-fold.

**Figure 3:**
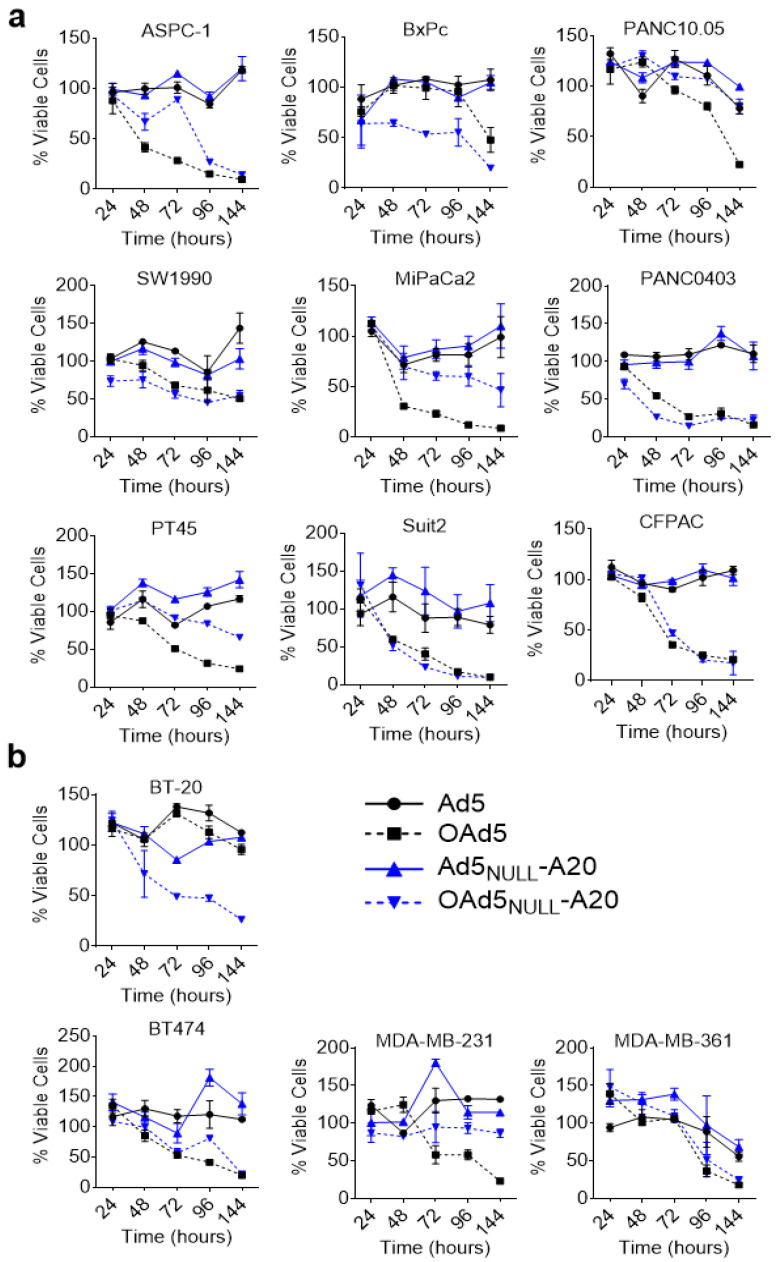
**Oncolytic activity of Ad5 and Ad5_NULL_-A20 in pancreatic and breast cancer cell lines:** Pancreatic cell lines (**a**) and breast cancer cell lines (**b**) were transduced with 5000 vp/cell of either oncolytic Ad5 (black squares), oncolytic Ad5_NULL_-A20 (blue triangle upside down), replication deficient Ad5 vector (black circle), or replication deficient Ad5_NULL_-A20 vector (blue triangle). Cell viability was quantified 48 h post infection using MTS assay. Error bars represent standard deviation of *n* = 4.

Cell killing by oncolytic virus, as gauged by MTS cell viability assay, also correlated well with the expression levels of αvβ6 integrin/CAR (Figure 3).

Oncolytic Ad5_NULL_-A20 was able to effectively kill αvβ6^high^/CAR^null^ BT-20 cells, which were resistant to oncolytic Ad5, due to the lack of expression of the Ad5 receptor, CAR. This was reversed in the αvβ6^null^/CAR^high^ MDA-MB-361 cell line, with only Ad5 showing cell killing. Cells expressing high levels of αvβ6 (BxPc, PANC0403, Suit2) were killed more efficiently by oncolytic Ad5_NULL_-A20 than by oncolytic Ad5.

**Figure 4:**
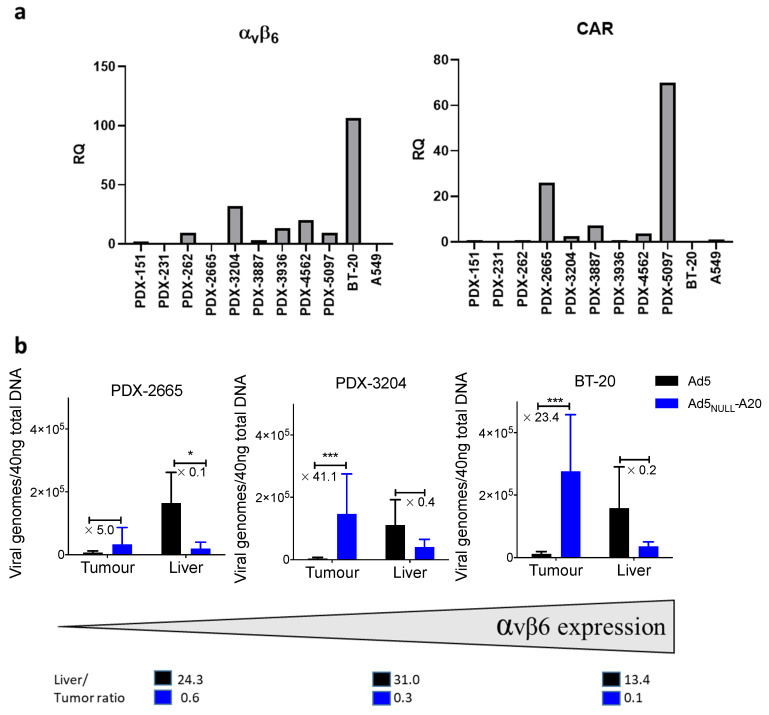
**Biodistribution of Ad5 and Ad5_NULL_-A20 in an in vivo breast cancer model**: A panel of PDX breast cancer lines (**a**) were analyzed by RT-qPCR to determine levels of αvβ6 and CAR, gene expression relative to A549 cell line. PDX and BT-20 tumors were established in NSG mice (**b**), when tumors reached a palpable size, mice were injected intravenously with 1 × 10^11^ vp of Ad5 (black bars) or Ad5_NULL_-A20 (blue bars). Forty-eight hours postinjection, organs were harvested, and qPCR for viral genomes was performed (* *p* < 0.05; *** *p* < 0.001). Liver/tumor ratios are included below the graphs.

Given the favorable, tumor-selective targeting observed in vitro, we performed in vivo studies to evaluate whether intravenous administration of replication-deficient Ad5_NULL_-A20 resulted in targeting to permissive tumors in vivo in NSG mice. We elected to use replication-deficient vectors to get an accurate reflection of viral biodistribution, since the use of oncolytic vectors would result in the replication of virus within the xenograft and would therefore skew the data towards increased accumulation in the tumor. Since PDX platforms have more translational relevance, we selected two PDX models of breast cancer from a panel available in-house, which were found to have low (PDX2665) or med/high (PDX3204) levels of αvβ6 integrin based on mRNA expression relative to A549 cells (Figure 4a), as well as a BT-20 (αvβ6^high^).

Forty-eight hours after viral injection, organs were harvested, and qPCR for viral genomes was performed on DNA isolated from the liver and tumors. Our data demonstrate that in all three models tested, Ad5_NULL_-A20 showed increased tumor accumulation compared to Ad5 (Figure 4b).

We found 5-fold (low αvβ6 model, PDX2665), 41-fold (for med αvβ6 model, PDX3204) and 23-fold (for high αvβ6 model, BT-20) higher amounts of viral DNA in the tumors of mice injected with Ad5_NULL_-A20 compared to mice injected with Ad5. We also noted decreased hepatic accumulation, leading to dramatically improved liver-to-tumor ratios. Improvements of over 100-fold in the liver-to-tumor ratio was seen in both the PDX3204 (31.0 vs. 0.3) and BT20 (13.4 vs. 0.1) models when comparing Ad5 to Ad5_NULL_-A20. qPCR for viral genomes was carried out 48 h after intravenous administration. Previous studies have shown rapid hepatic sequestration and degradation of unmodified Ad5 capsids within the first 24 h [36] following administration mediated by Kupffer cells [37,38]. qPCR measurements taken at 48 h, as here, will underestimate initial uptake of unmodified Ad5 by the liver, and future studies at earlier timepoints will be required to appropriately dissect out the effects that the capsid modifications in Ad5_NULL_-A20 have on early sequestration by the liver.

## 4. Discussion

We previously generated a triple-detargeted Ad5-based virotherapy (Ad5_NULL_) that could be specifically retargeted to αvβ6 integrin-expressing cells by incorporation of the A20 peptide, and demonstrated this to be an effective treatment in an in vivo model of peritoneal ovarian cancer [20]. Here, we have progressed development of Ad5_NULL_-A20 to evaluate its potential in other cancers with high unmet clinical need where αvβ6 integrin has previously been reported to be commonly expressed. Analysis of a large number of pancreatic ductal adenocarcinoma (PDAC, 383 primary tumors, 7 lymph node, and 8 distant metastases) and 34 pancreatic intraepithelial neoplasia (PanIN) specimens revealed a high prevalence of αvβ6-integrin expression in PDAC primaries (88%) and in almost all metastases, as well as in PanIN (57%) [39]. Further, an analysis of 2000 breast cancer patient samples [27] showed high expression of αvβ6 integrin in 15% to 16% of invasive ductal carcinoma and a significant association between high expression of αvβ6 integrin and poor survival. Pancreatic and breast cancers were therefore considered good targets for Ad5_NULL_-A20 virotherapy. We showed αvβ6 was expressed in seven of nine pancreatic cancer cell lines and three of four breast cancer cell lines tested. Furthermore, Ad5_NULL_-A20 was able to selectively infect and kill pancreatic and breast cancer cell lines in an αvβ6 dependent manner. Cell lines with high αvβ6 integrin showed enhanced oncolytic cell killing compared to Ad5.

Whilst intratumoral injection of oncolytic vectors has been effective in cancer treatments [40], development of oncolytic vectors that can be given systemically are required for treatment of nonaccessible tumors. Various strategies have been employed to improve systemic of delivery of Ads, including shielding of the virus [41] and swapping of hypervariable loops with non-FX-binding serotypes to reduce liver transduction [42]. Here, we demonstrate that Ad5_NULL_-A20 can effectively target αvβ6-positive tumors established in NSG mice following intravenous injection. Even the low-αvβ6-expressing PDX 2665 showed increased tumor accumulation compared to Ad5, presumably due to the increased bioavailability of this viral vector since its modification reduces off-target depletion in sinks such as the liver and spleen, thus maximizing the likelihood of passive, as well as active, accumulation within the tumor microenvironment. We saw a 40× increase in tumor accumulation in the med αvβ6 PDX 3204 model compared to Ad5, coupled with a substantial decrease in liver accumulation. This manifested in an improvement in liver-to-tumor ratio of over 100-fold compared to Ad5. Further studies such as PET imaging and sampling of organs within 30 min are required to investigate liver biodistribution at early timepoints. It is also currently unclear how interactions with blood clotting factors influence early uptake of adenovirus by Kupffer cells, with some studies showing ablation of FX binding reduces innate immune response to Ad5 [43]. The high-αvβ6-BT-20 model showed the highest accumulation of Ad5_NULL_-A20, as expected. However, accumulation of Ad5 was higher than the PDX models, despite the lack of CAR in BT-20 cells. The accumulation of Ad5_NULL_-A20 was still over 20× higher than Ad5 and liver-to-tumor ratio improved over 100×.

## 5. Conclusions

Ad5_NULL_-A20 represents an exciting platform with significant potential to treat αvβ6-integrin-expressing tumors by both intraperitoneal [20] and, as demonstrated here, systemic approaches. This heavily engineered virotherapy platform has the potential to be further armed with therapeutic transgenes, offering the enticing possibility that the platform can be adapted to enable the overexpression of potent, virally mediated immunological transgenes within the tumor microenvironment following systemic administration. The Ad5_NULL_-A20 platform therefore has significant potential for efficacy and onward clinical translation.

## 6. Patents

Cardiff University has patented the Ad5_NULL_ platform and the Ad5_NULL_-A20 targeted virotherapy (WO201958914A1).

## Data Availability

Due to confidentiality agreements with research collaborators, supporting data can only be made available to bona fide researchers subject to a nondisclosure agreement. Details of the data and how to request access can be made via the corresponding author.
